# Effects of different artificial diets on commercial honey bee colony performance, health biomarkers, and gut microbiota

**DOI:** 10.1186/s12917-022-03151-5

**Published:** 2022-01-21

**Authors:** Vincent A. Ricigliano, Steven T. Williams, Randy Oliver

**Affiliations:** 1 USDA-ARS, Honey Bee Breeding, Genetics, and Physiology Research, Baton Rouge, Louisiana 70820 USA; 2 ScientificBeekeeping.com, Grass Valley, California 95945 USA

## Abstract

**Background:**

Honey bee colonies managed for agricultural pollination are highly dependent on human inputs, especially for disease control and supplemental nutrition. Hives are routinely fed artificial “pollen substitute” diets to compensate for insufficient nutritional forage in the environment. The aim of this study was to investigate the effects of different artificial diets in a northern California, US commercial beekeeping operation from August through February. This time period represents an extended forage dearth when supplemental nutrition is used to stimulate late winter colony growth prior to almond pollination in the early spring. A total of 144 honey bee colonies were divided into 8 feeding groups that were replicated at three apiary sites. Feeding groups received commercial diets (Global, Ultra Bee, Bulk Soft, MegaBee, AP23, Healthy Bees), a beekeeper-formulated diet (Homebrew), or a sugar negative control. Diets were analyzed for macronutrient and amino acid content then evaluated with respect to honey bee colony population size, average bee weight, nutrition-related gene expression, gut microbiota abundance, and pathogen levels.

**Results:**

Replicated at three apiary sites, two pollen-containing diets (Global and Homebrew) produced the largest colonies and the heaviest bees per colony. Two diets (Bulk Soft and AP23) that did not contain pollen led to significantly larger colonies than a sugar negative control diet. Diet macronutrient content was not correlated with colony size or health biomarkers. The sum of dietary essential amino acid deficiencies relative to leucine content were correlated with average bee weight in November and colony size used for almond pollination in February. Nutrition-related gene expression, gut microbiota, and pathogen levels were influenced by apiary site, which overrode some diet effects. Regarding microbiota, diet had a significant impact on the abundance of *Bifidobacterium* and *Gilliamella* and trended towards effects on other prominent bee gut taxa.

**Conclusions:**

Multiple colony and individual bee measures are necessary to test diet efficacy since honey bee nutritional responses are complex to evaluate. Balancing essential amino acid content relative to leucine instead of tryptophan may improve diet protein efficiency ratios. Optimization of bee diets could improve feed sustainability and agricultural pollination efficiency by supporting larger, healthier honey bee colonies.

**Supplementary Information:**

The online version contains supplementary material available at 10.1186/s12917-022-03151-5.

## Background

Honey bees (*Apis mellifera*) are important agricultural pollinators that are also maintained for their honey and other hive products. Commercially managed bee colonies may be transported over long distances for pollination services and for access to nutritional forage, exposing them to a variety of biotic and abiotic stressors. The US beekeeping industry is experiencing annual colony losses that are on average twice as high as historical records [1]. These losses are attributed to variety of interacting stressors such as pathogens [2] and pesticides [3], but malnutrition is an increasing threat that synergizes with other factors [4]. A number of sub-lethal effects are correlated with poor nutrition including impaired immune function and increased susceptibility to disease and agrochemicals [5–11].

Abundant floral resources are crucial for honey bee brood production, immune function, and overwintering survival [12–17]. Nectar serves as a carbohydrate source while pollen is the sole source of proteins, lipids, and micronutrients [13]. Pollen nutrition is of particular concern to modern beekeeping for a number of reasons. First, pollen stimulates colony growth and confers resilience to environmental stressors by replacing bees that may be dying at accelerated rates. Second, pollen stores in a colony can quickly deplete due to inclement weather or poor foraging conditions. Pollen must therefore be available in sufficient quantities throughout the brood producing season to support population growth. Lastly, varied flower sources are necessary to meet bee nutritional requirements since the composition of pollen varies  primarily according to plant species [18, 19]. Intensively cultivated landscapes are associated with reduced floral diversity and reduced nutritional value [20–22]. Climate change also poses threats to bee nutrition. Plant responses to climate change, which include altered flower, nectar, and pollen production, will change floral resource availability with potentially dire consequences for plant-pollinator networks [23–25].

Beekeepers feed artificial “pollen substitute” diets to offset periods of inadequate pollen forage and to increase colony strength prior to pollination services [26–29]. A number of commercially available and beekeeper-formulated diets are used in beekeeping operations throughout the United States. For instance, 87% of US beekeepers claim to feed supplemental nutrition (Bee Informed Partnership, National Management Survey, https://bip2.beeinformed.org/survey/). The protein content of pollen is vital for the colony and has been a major focus of artificial diet development. In particular, the amino acids arginine, histidine, isoleucine, leucine, lysine, methionine, phenylalanine, threonine, tryptophan, and valine are considered essential for honey bees [30]. Pollen also contains essential lipids that are important to various aspects of bee physiology [31–33]. Different combinations of ingredients have been used as a partial or full replacement for natural pollen. Artificial bee diets commonly incorporate protein-rich ingredients such as soy, pea, yeast, casein, egg, and microalgae. Some diets include a fraction of bee-collected pollen, which has been shown to increase consumption and brood rearing [34, 35]. Diet effectiveness varies according to nutrient composition and test conditions, especially when compared to natural pollen.

Artificial diets are typically fed to bee colonies as a dough-like sugar patty. Feed is placed inside the hive for young worker bees to consume and build up nutrition stores that are used to rear brood. Hence, colony population size and worker bee physiology are important metrics for assessing diet efficacy. Beekeepers and farmers use colony population size to negotiate pollination contracts because larger colonies lead to increased pollination efficiency [36, 37]. Researchers use field-collected bee samples to obtain information on the quality of nutritional resources available to bee colonies [38–40]. Measures of worker bee head and thorax weight are indicators of nutrient assimilation into brood food-producing head glands and flight muscles, respectively. Molecular biomarkers such as mRNA expression of the storage protein vitellogenin (vg) have been used to assess bee nutritional status because vg levels are linked to diet quality [16, 40–43]. Nutrition also induces changes in the honey bee gut microbiota, with consequences on host immune function and pathogen susceptibility [44–47]. Microbiota abundance is therefore a potential biomarker of honey bee disease and nutritional status that warrants further investigation.

Supplemental feeding is a management strategy with significant financial and labor costs for large-scale beekeepers. The purpose of this study was to compare the effects of different artificial diets on commercial honey bee colonies in northern California, US during an extended forage dearth. Colonies were subjected to feeding regimens that were replicated across three apiary sites. We monitored colony population size as well as physiological and molecular biomarkers in field-collected bee samples. We hypothesized that (1) colonies fed protein diets would have larger populations and elevated health biomarkers, (2) diets containing natural pollen would have a greater positive impact than completely artificial diets and (3) colony performance and health would be influenced by the local environments of different apiary sites.

## Methods

### Experimental setup and honey bee colony management

In July 2019, one hundred and forty four honey bee colonies were established via splits of healthy parent colonies sourced from Golden West Bees (Grass Valley, CA), which managed all colonies in this study. Colonies were maintained in identical double deep Langstroth hives. All colonies were headed with first-year queens produced by Golden West Bees (Grass Valley, CA). All queens were of the same stock and grafted from the same mother.

**Fig. 1:**
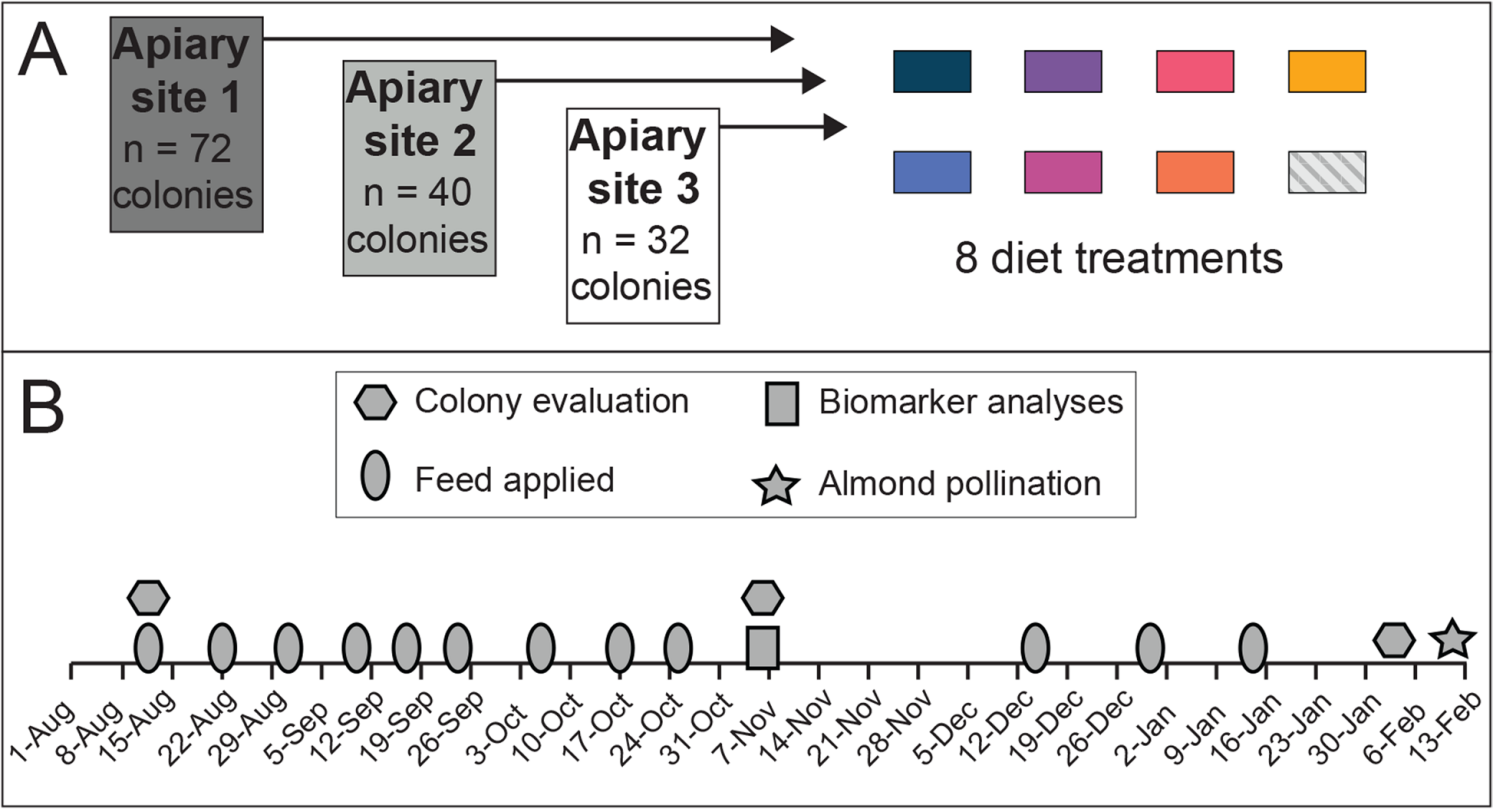
Schematic overview of the **A)** experimental design and **B)** experimental timeline.

Three apiary sites were chosen because they have historically exhibited a lack of natural forage during the experimental time period. Apiary site coordinates were as follows: 39.12329, −121.12924 (apiary site 1), 39.09106, −121.09405 (apiary site 2), and 39.17854, −120.99174 (apiary site 3). At the start of this experiment in August 2019, colonies were blocked according to population size at each apiary site, and randomly assigned to diet treatment groups (Fig. 1A). Colonies were fed either a sugar negative control diet or one of seven protein diets. Two diets, Global (Global Patties, Airdrie, AB, Canada) and Homebrew, contained 15 and 20% natural pollen, respectively. The remaining diets were fully artificial diets that generally contained mixtures of plant-based proteins with no pollen: Ultra Bee (Mann Lake Ltd., Hackensack, MN, USA), Bulk Soft (Mann Lake Ltd., Hackensack, MN, USA), MegaBee™ (Castle Dome Solutions, Helena, AR, USA), AP23 ® (Dadant, Hamilton, IL, USA), and Healthy Bees™ (Healthy Bees LLC, Upper Sandusky, OH, USA). Diets were fed according to the timeline outlined in Fig. 1B. Approximately 450 g of diet was applied between the upper and lower box in each colony and unconsumed food was measured prior to the application of fresh patties. Colony population size was measured in August, November, and February by counting the number of frame interspaces within a hive that were filled with bees (Fig. 1B).

In November 2019, a representative subset of colonies were sampled for physiological and molecular analyses (Fig. 1B). Bee samples were obtained from 3–4 randomly selected colonies in each treatment group at each apiary site (*n* = 9–12 colonies per diet treatment). Bees were collected from the top bars proximal to diet patties to represent a cohort of workers engaged in feed consumption and nutrient assimilation. All samples were vacuum-collected into 50 ml conical tubes which were immediately frozen on dry ice, and stored at −80 °C for further processing.

### Nutritional analyses of honey bee diets

Diets were analyzed for nutrient compositions by Minnesota Valley Testing Lab, Inc, (New Ulm, MN, USA) according to the Official Methods of Analysis of the Association of Official Analytical Chemists (AOAC) [48] and the Approved Methods of the American Association of Cereal Chemists (AACC) [49]. The following analyses were performed: crude protein by AOAC method 990.03, crude lipid by AOAC method 2003.05, fiber by AOAC method 978.10, amino acids by AOAC method 994.12, sugars by AACC Method 80–04.

### Bee weight measures and RNA extractions

Field-collected bee samples were dissected on dry ice and separated into head, thorax (excluding legs and wings), and abdomens. For each colony, parts were separately pooled into 15 heads, 15 thorax, and 30 abdomens. Head and thorax weights were determined by drying at 60 °C to a constant weight and recording to the nearest 0.1 mg. Pools of bee abdomens were homogenized in 2 ml of Maxwell® simplyRNA homogenization solution (Promega, Madison, WI, USA) using a Bead Rupture Elite bead mill (OMNI International, Kennesaw, GA, USA). Samples were centrifuged and 100 μl of the supernatant was removed for RNA extraction with a Monarch® total RNA miniprep kit (New England BioLabs, Ipswich, MA, USA) according to the manufacturer’s instructions.

### Quantitative PCR (qPCR)

Gene expression of honey bee vitellogenin as well as gut microbiota and pathogen abundances were measured by quantitative PCR (qPCR) using cDNA template generated from total RNA. For gut microbiota abundance, samples were screened with primers targeting the bacterial 16S rRNA gene of five prominent taxa: *Lactobacillus* Firm 5, *Lactobacillus* Firm 4, *Bifidobacterium*,

*Gilliamella*, and *Snodgrassella* (Table S1). Colony samples were screened for pathogens using primers targeting Deformed Wing Virus (DWVA) and the *Nosema ceranae* rRNA gene. All qPCR reactions were performed in triplicate as follows: initial denaturation at 95 °C for 5 min; 40 cycles with denaturation at 95 °C for 15 s; and a primer-pair-specific annealing and extension temperature (Table S1) for 30 s. The reactions were carried out using SsoAdvanced Universal SYBR® Green Supermix (Biorad, Hercules, CA, USA) in triplicate on an CFX96TM Real-Time PCR Detection System (Biorad, Hercules, CA, USA). To confirm the absence of contaminating genomic DNA and primer dimers in the assay, we tested amplification and melting curves in negative controls consisting of DNase-treated total RNA without reverse transcriptase. Relative transcript levels were determined based on standardized Ct values (Δ Ct) using β-actin for normalization [50].

### Statistical analyses

Effects of diet, apiary site, and diet * apiary site were evaluated using mixed-model ANOVA with post hoc contrasts of least squares mean differences as required. Dependent variables were evaluated for normality using fit statistics and probability plots. Variables with deviations from normality were re-evaluated after log transformation. All analyses were conducted in JMP v11 and Prism v7.

## Results and discussion

Honey bee colonies are fed artificial diets to offset a lack of nutritional pollen forage in the environment. Supplemental feeding is expected to stimulate brood production and colony population growth, especially leading up to pollination services when beekeepers are compensated based on colony size. Despite the widespread use of nutrition supplements, benefits of feeding artificial diets vary in large-scale beekeeping operations. This study tested the effects of different artificial diets in an arid, forage-deficient region in northern California, from August through early February. This time period represents an extended forage dearth when supplemental nutrition is used to stimulate late-winter growth prior to almond pollination in the early spring. Diets were evaluated based on honey bee colony population size, average bee weight, nutrition-related gene expression, gut microbiota abundance and pathogen levels.

### Nutrient composition of honey bee diets

Honey bee artificial bee diets incorporate protein-rich ingredients to replicate or extend pollen nutrition. Some commercial diets appear to match the effectiveness of natural pollen for limited periods of time [51, 52], however many of these products have not been robustly tested. We chose to compare different commercial diets (Ultra bee, Global, Bulk Soft, Mega Bee, AP23, and Healthy Bees) based on popular use in US beekeeping operations. Since many operations mix their own feed, we also tested a beekeeper-derived formulation (Homebrew). Diet information is listed in Table S2, although the complete formulas were proprietary. In general, the diets contained a mixture of plant proteins, sugar, vitamins and minerals, and plant essential oils. The Global and Homebrew diets were considered semi-artificial as they contained 15 and 20% natural pollen, respectively (Table S2).

**Fig. 2:**
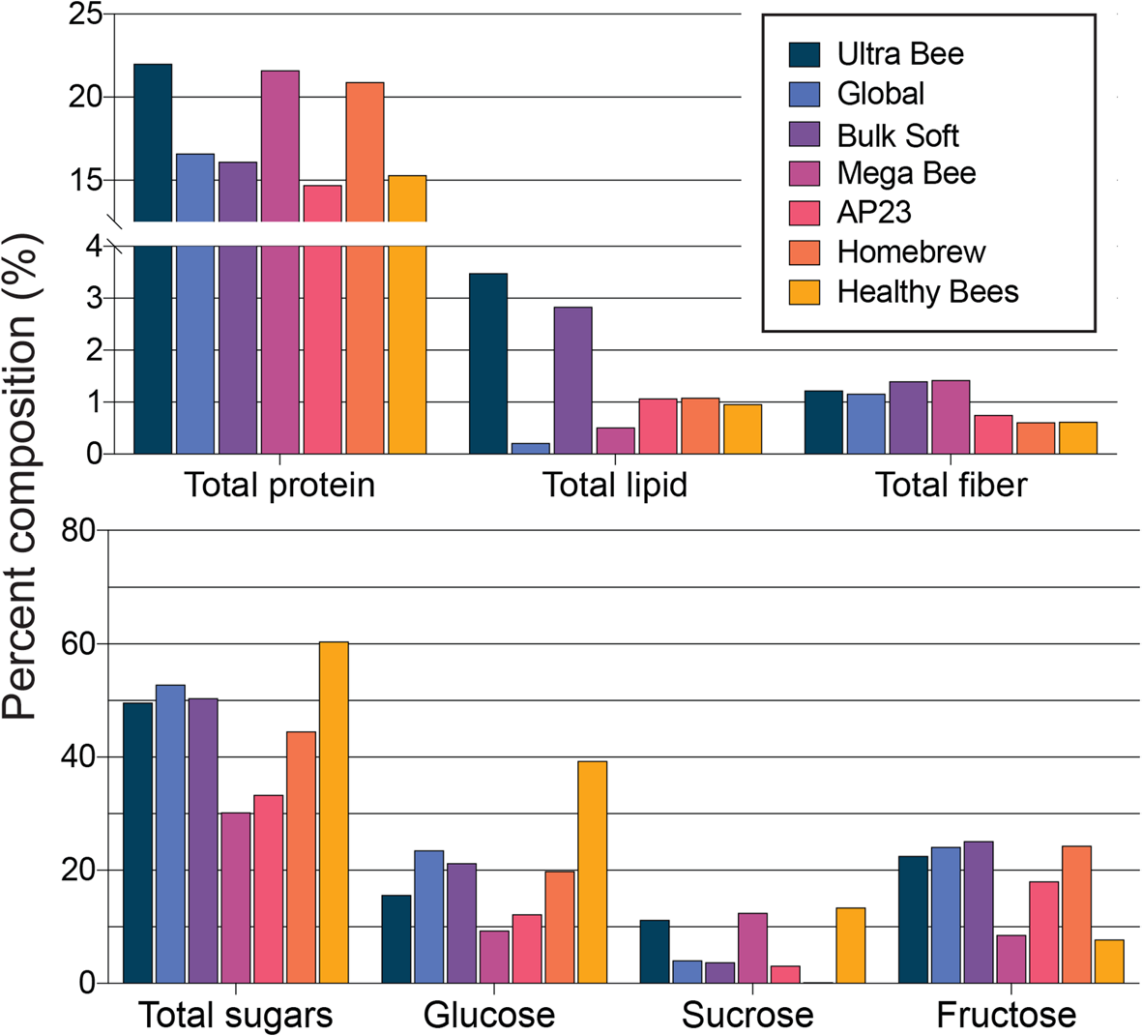
Macronutrient content of the different honey bee diets.

Pollen is the sole source of natural protein for honey bees. Protein obtained from pollen is central to bee development, and immunity, and stress responses [13, 53]. Previous analyses of pollens ranged from 2–60% crude protein [18]. A protein content of 20% has been recommended as the minimum for a diet to be functional under laboratory conditions [53] although bee colonies can grow on natural pollen with lower protein concentrations. Crude protein content of the tested diets were as follows: Ultra bee (22.0%), Mega Bee (21.6), Homebrew (20.9%), Global (16.6%), Bulk Soft (16.1%), Healthy Bees (15.3%), AP23 (14.7%) (Fig. 2).

Recent focus on dietary lipids has revealed important functional roles in honey bees [54, 55]. However, dietary lipid requirements for honey bees are not fully understood and are confounded by the fact that insects can synthesize lipids from carbohydrates [56]. Crude lipid content was highest in the Ultra Bee (3.5%) and Bulk Soft (2.8%) diets, followed by AP23, Homebrew, and Healthy Bees (~1% each) while lipid content was lowest in the Global diet (0.2%) (Fig. 2). Previous analyses of pollen lipid content ranged from 1 to 20% depending on plant species [19]. Overall, diet lipid contents fell within the lower range of values reported for natural pollen.

Crude lipid measures do not take into account the subclasses of lipids that are important to various aspects of bee physiology. Pollen contains a diversity of lipid types such as phospholipids, fatty acids, and sterols [57, 58]. Dietary fatty acids are incorporated into cellular macromolecules such as membrane lipids and lipoproteins [33]. Linoleic acid and alpha-linoleic acid are two fatty acids that are essential for animals [59], including bees [31, 32]. Further quantification of artificial diet’s lipid classes can provide additional information on their specific composition and nutritional value [43].

Dietary fiber can modulate animal microbiota and host animal physiology [60]. The diets had similar crude fiber contents of <1.4% (Fig. 2). Nearly 10-fold higher levels of dietary fiber were determined in different pollens, which ranged from 11 to 16% [61].

A mixture of sugars is well accepted by bees [62] and supports a favorable diet texture and moisture content due to the hygroscopicity of fructose. Total sugar content was highest in the Healthy Bees diet (60.4%) and lowest in the Mega Bee diet (30.2%). Ultra bee, Global, and Bulk Soft diets were approximately 50% sugar. Glucose and fructose accounted for the majority of sugars in all diets (Fig. 2).

**Fig. 3:**
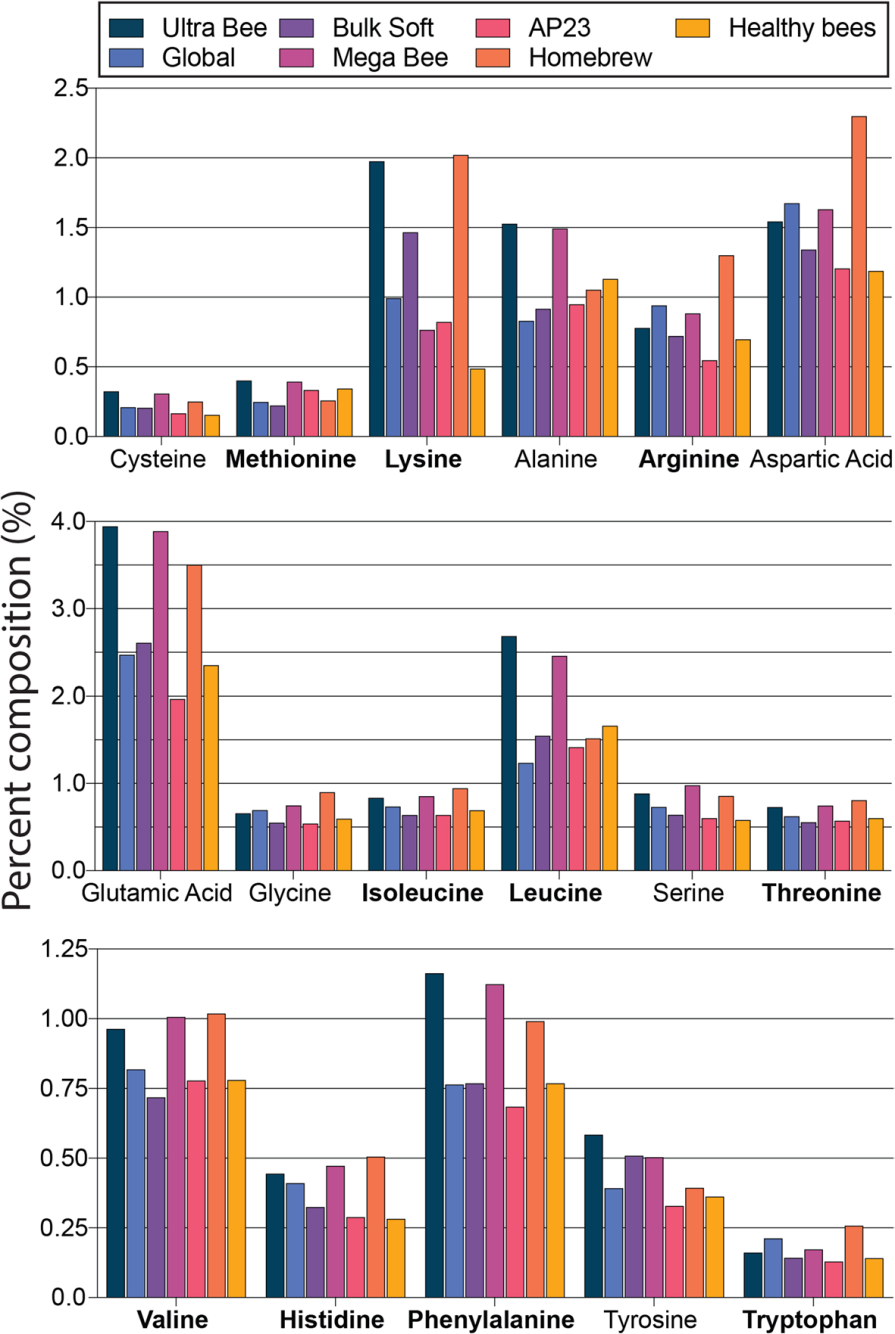
Amino acid content of the different honey bee diets. Essential amino acids are emphasized in bold.

To further investigate the quality of dietary proteins, complete amino acid profiles were obtained (Fig. 3). The following amino acids are considered essential for honey bees and must be present in the diet: arginine, histidine, isoleucine, leucine, lysine, methionine, phenylalanine, threonine, tryptophan, and valine. The greatest proportional requirements are for leucine, followed by isoleucine and valine [30]. According to Liebig’s Law, the rate of protein synthesis achieved by a given diet is determined by the concentration of the essential amino acid (EAA) present in the smallest proportion with respect to the animal’s requirements [63]. Since leucine is the predominate EAA required for honey bee growth [30], it is a valid reference point from which to analyze diets for EAA balance. Based on this interpretation of [30], leucine should therefore constitute 16% of total EAAs in an optimal diet (Fig. S1A). The leucine content relative to total EAAs for the tested diets were as follows: Homebrew (15%), Global (16%), Bulk Soft (19%), AP23 (21%), Ultra Bee (23%), Healthy Bees (23%), and Mega Bee (24%) (Fig. S1B).

### Colony performance

At the start of the experiment in August, the 144 honey bee colonies were evaluated then assigned feeding groups that were replicated across three apiary sites (Fig. 1A). The use of multiple apiary sites is prerequisite for understanding the impacts of hive manipulations since colony performance and health are strongly influenced by local environments [21, 39]. Following a feeding period of 84 days, during which colonies depended upon the diets as their main source of nutrition, colonies were evaluated in November. Feeding regimens paused during a one-month winter broodless period, and then resumed again in early December (Fig. 1B). Finally, colonies were evaluated in February prior to their use in almond pollination services.

**Fig. 4:**
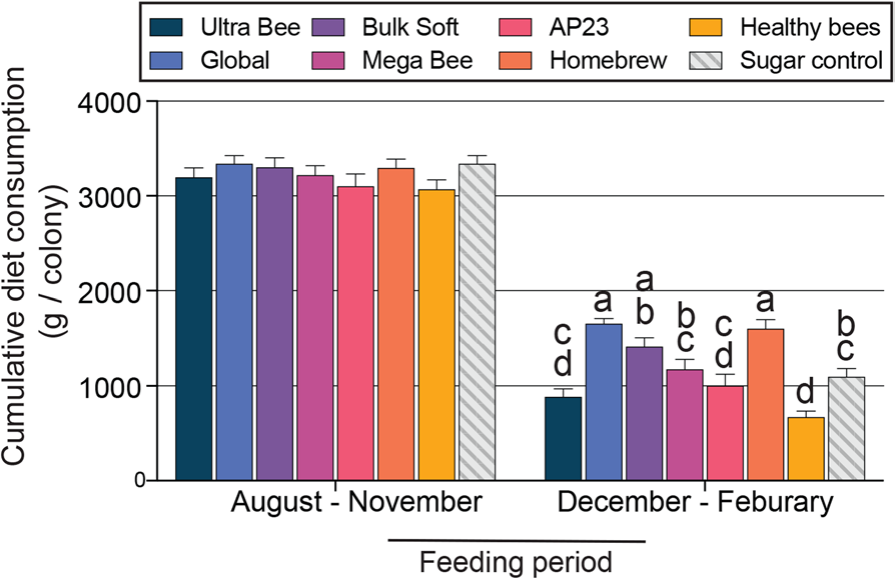
Cumulative diet consumption by honey bee colonies during August–November and December–February feeding periods. Error bars represent standard error (SE). For each feeding period, columns with different letters are significantly different at α = 0.05.

Diet consumption was not significantly different during the August to November feeding period (*P* > 0.05), but consumption was significantly different during the December to February feeding period (F _7, 135_ = 14.4, *P* < 0.0001; Fig. 4). During this time period, Homebrew and Global diets had the highest consumption whereas the Healthy Bees diet had the lowest consumption (Fig. 4).

**Fig. 5:**
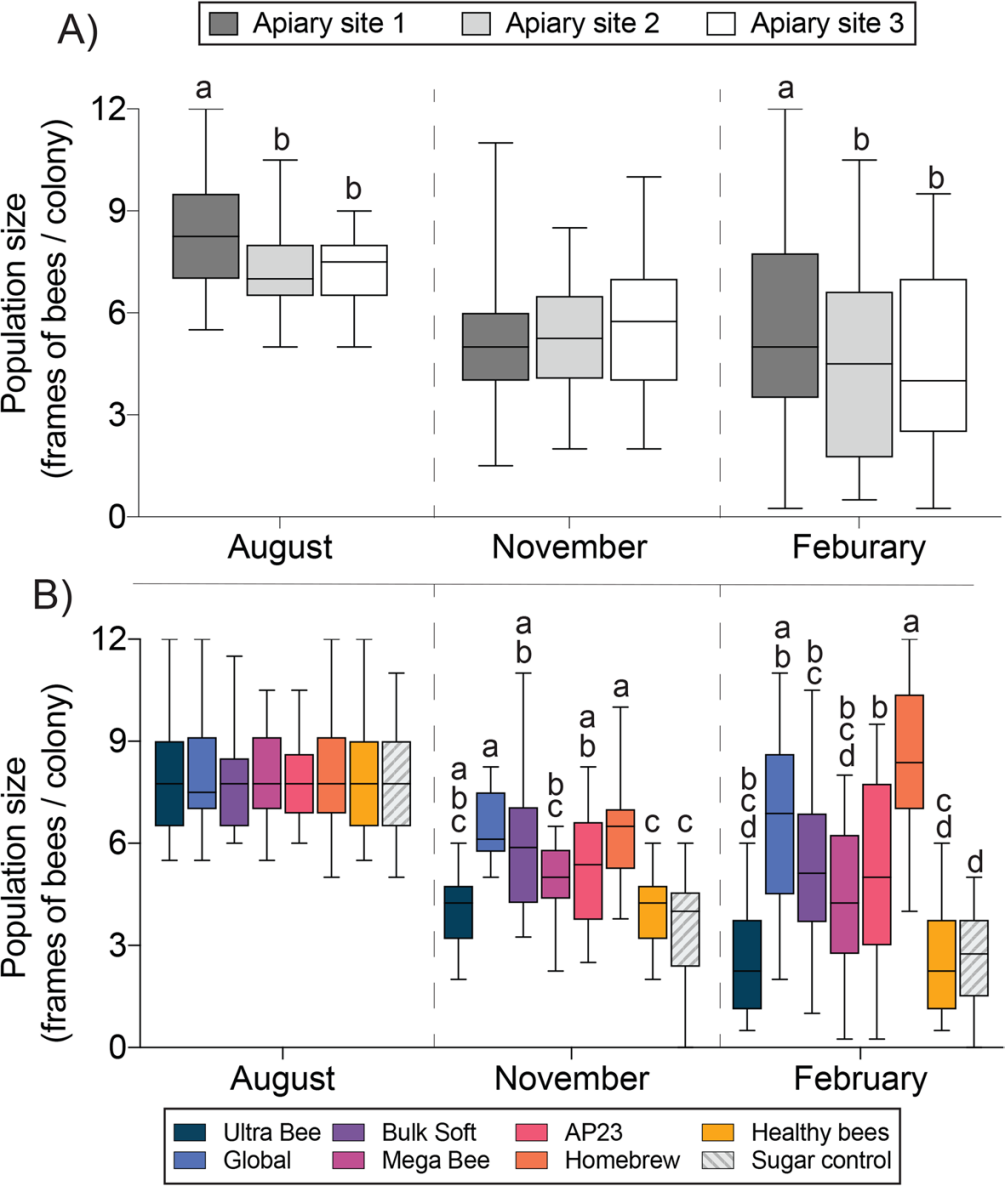
Honey bee colony population sizes evaluated at the August, November and February time points. **A** Colony population size at each apiary site. **B** Colony population size according to diet treatment. Plot whiskers show minimum and maximum values with the lower and upper edge of each box denoting the 25th to 75th percentiles and median as a horizontal bar. At each time point, boxes with different letters are significantly different at α = 0.05.

Apiary site had a significant effect on colony size in August (F _2, 142_ = 6.3, *P* = 0.0023). Colonies at apiary site 1 had an average of 8.3 frames of bees, whereas apiary sites 2 and 3 averaged 7.4 frames of bees (Fig. 5A). The treatment groups were assigned in August and no treatment effects were detected because starting colony size was blocked for each apiary site. Apiary site did not impact colony size in November (*P* > 0.05) but feeding treatment had a significant effect (F _7, 135_ = 9.0, *P* < 0.0001). Global and Homebrew diets produced the largest colonies with an average of 6.4 frames of bees. Healthy Bees and sugar control diets produced the smallest colonies, averaging 3.9 and 3.7 frames of adult bees, respectively (Fig. 5B). Apiary site had a significant effect on colony size in February (F _2, 128_ = 4.9, *P* = 0.0092). Colony size at each apiary site was as follows: site 1 (5.6 frames of bees/colony) > site 2 (4.4 frames of bees/colony) = site 3 (4.3 frames of bees/colony) (Fig. 5A). Feeding treatment had a significant effect on colony size in February (F _7, 123_ = 10.6, *P* < 0.0001). The Homebrew diet produced the largest colonies with an average of 8.1 frames of bees whereas the Healthy Bees diet produced the smallest colonies with an average of 2.2 frames of bees (Fig. 5B).

Diet macronutrient content did not appear to be a major factor in colony performance. The minimum amount of protein for a diet to be functional was previously determined to be 20 to 30% [53]. According to this criteria, Ultra Bee, AP23, and Homebrew had sufficient protein whereas the other diets did not. We found that diets with low protein contents produced larger colonies, which points to other factors driving diet effects. Similarly, diets with low lipid contents produced larger colonies. This result was consistent with the observation that a bee colony can increase in population size when fed a pollen diet from which lipids had been removed via extraction [64].

Under laboratory conditions, high protein to lipid (P:L) ratios have been shown to negatively impact bee physiology while low P:L ratios appear to have positive effects [54]. P:L ratios were not likely a major factor in colony performance since Homebrew and Global diets produced the strongest colonies but had high P:L ratios of 19:1 and 79:1, respectively (Fig. S2).

**Fig. 6:**
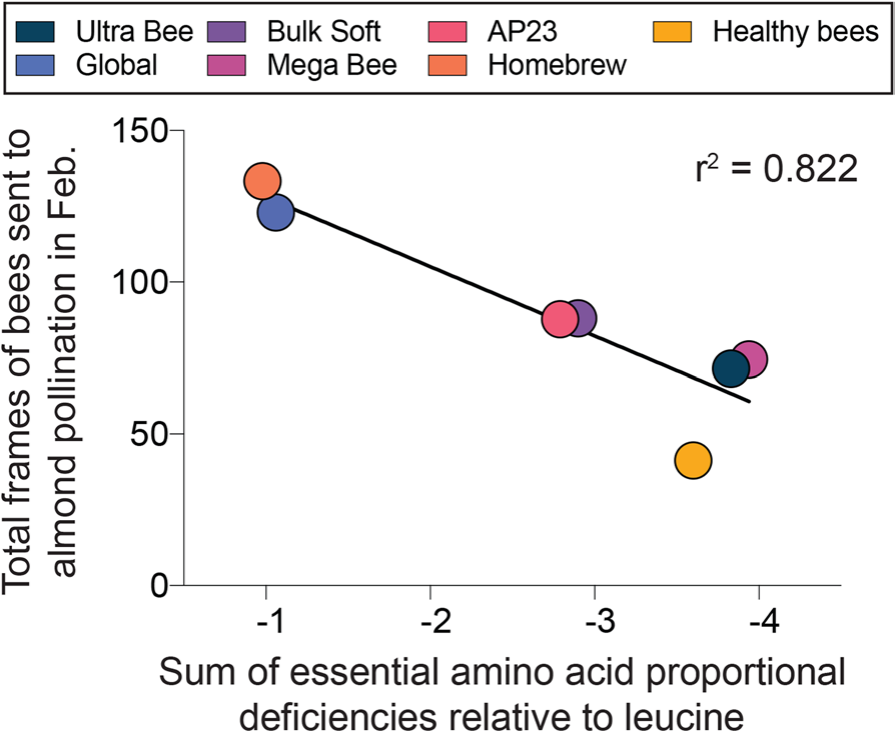
Relationship between the total frames of bees sent to almond pollination in February and the sum of dietary essential amino acid proportional deficiencies relative to leucine.

Since leucine is required in the highest proportion for honey bee growth [30], normalization of dietary EAA content relative to leucine revealed that some diet effects might be attributable to EAA deficiencies (Fig. S3). The sum of dietary EAA deficiencies relative to leucine was significantly correlated with the number of frames of bees that were sent to almond pollination in February (F _1,5_ = 23.16, *P =* 0.0048; Fig. 6). This result suggests that optimization of artificial diet EAAs proportional to leucine could improve colony growth via increased protein synthesis and leucine utilization.

### Honey bee nutritional status

Analysis of field-collected bee samples can provide physiological information about colonies subjected to different environments and management practices [38–40, 65]. During November colony evaluations, bee samples were collected from a random subset of colonies in each treatment group and at each apiary site (n = 9–12 colonies per diet treatment). Physiology of individual bees can fluctuate dramatically due to environmental factors, seasonal colony demography, and pathogen loads [65]. We used a pooled sampling approach to overcome individual bee variation and better represent colony-level physiological status. Bee samples were obtained adjacent to the diet patties since this spatial cohort of worker bees engages in food consumption and nutrient assimilation [66, 67]

**Fig. 7:**
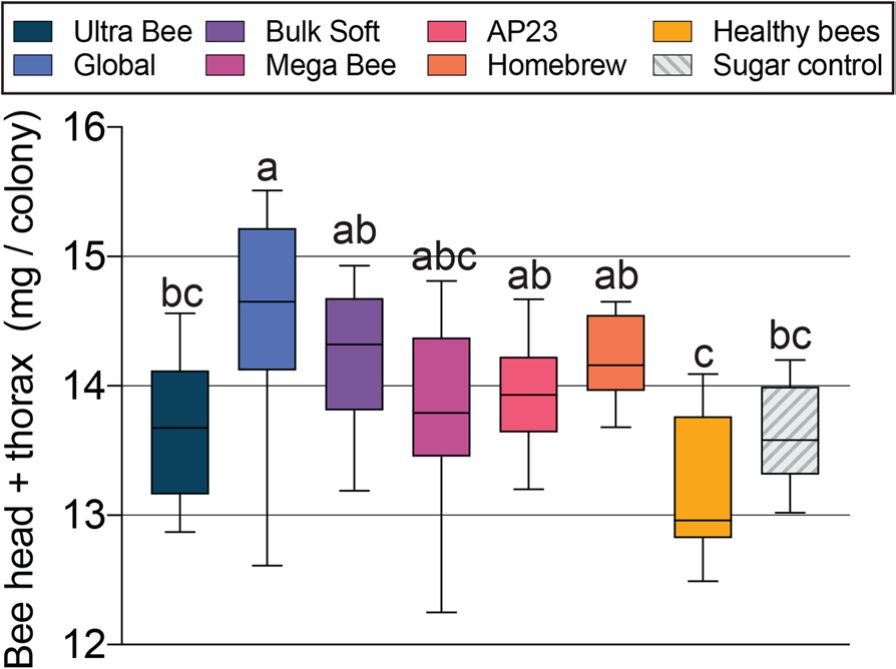
Combined bee head and thorax weights from colonies fed the different diets (n = 9–12 colonies per diet treatment). Plot whiskers show minimum and maximum values with the lower and upper edge of each box denoting the 25th to 75th percentiles and median as a horizontal bar. Boxes with different letters are significantly different at α = 0.05.

**Fig. 8:**
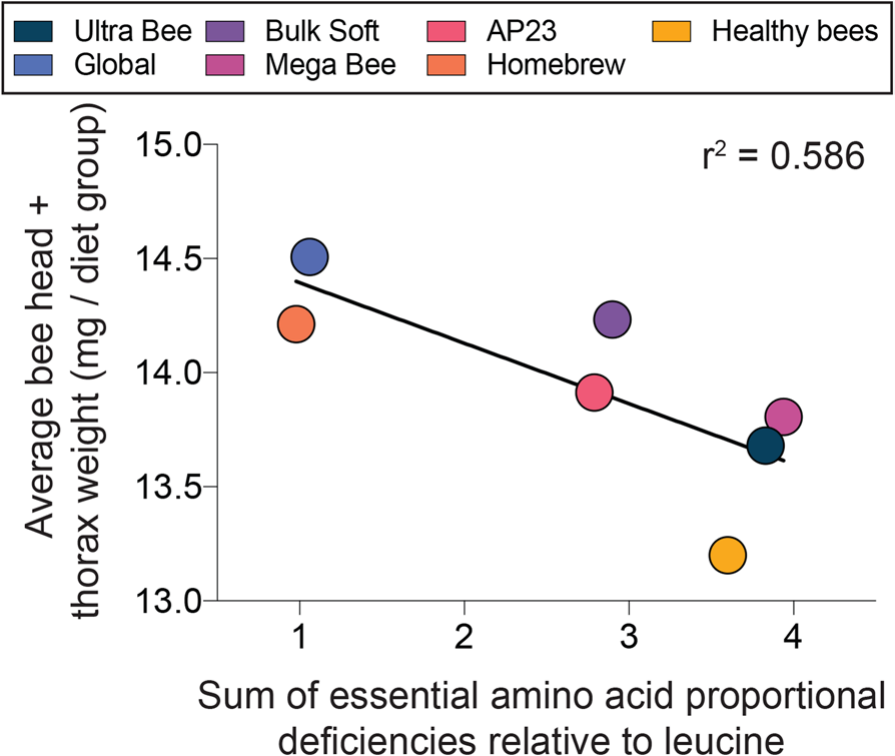
Relationship between average bee weight per diet group and the sum of dietary essential amino acid proportional deficiencies relative to leucine.

Higher head and thorax weights respectively reflect increased head gland development and flight muscle mass, bee attributes that are vital to colony fitness [14, 51, 66, 68]. The average combined head and thorax weight per colony was determined using pools of field-collected bees. Diet treatment had a significant effect on bee weights (F _7, 81_ = 6.3, *P* < 0.0001) but apiary site did not. In general, bee weights reflected colony population sizes but only the Global diet produced bees that were statistically heavier than the control (Fig. 7). Bee weight in November was significantly correlated with dietary EAA deficiencies relative to leucine (F _1,5_ = 7.07, *P =* 0.0449; Fig. 8). Taken together with effects on colony size, this result suggests that dietary EAA balance is important for individual bee development, which likely translates to improved colony growth.

**Fig. 9:**
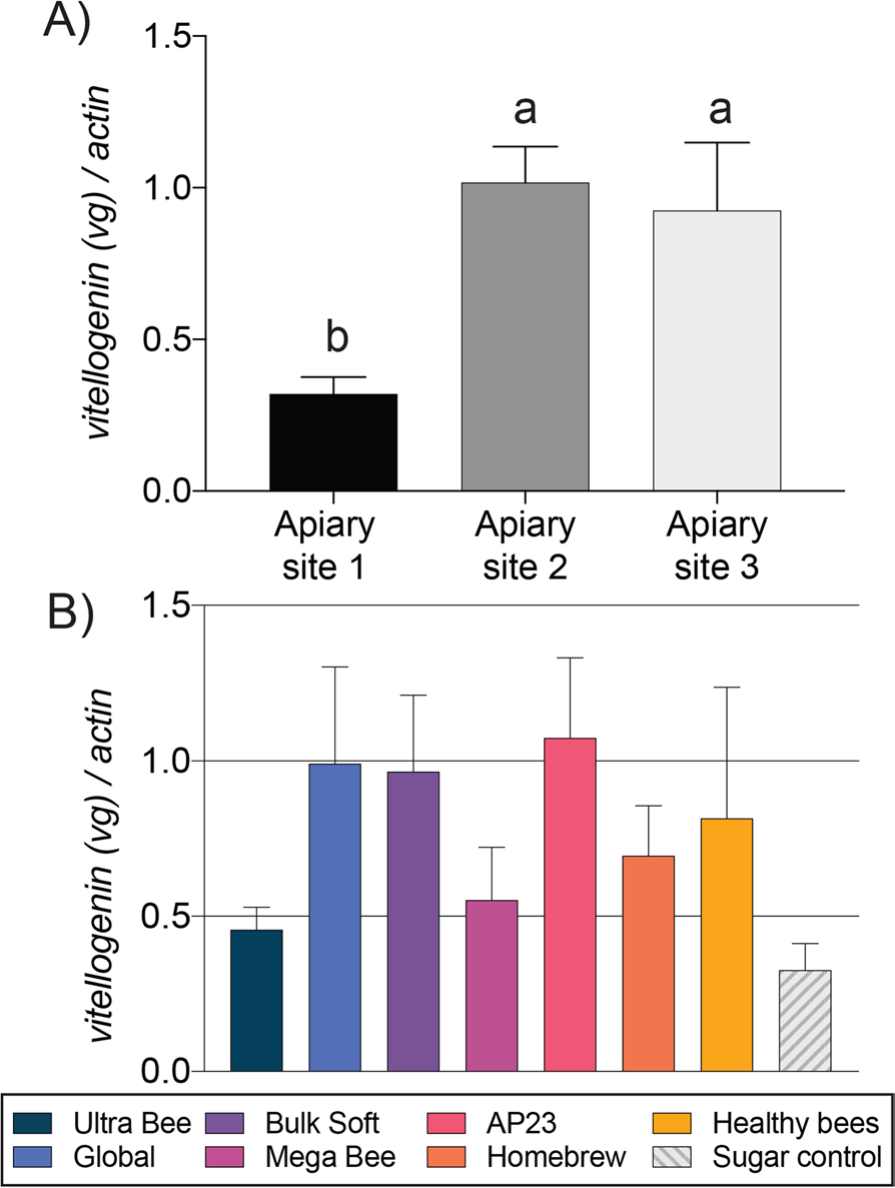
Relative mRNA expression of *vitellogenin,* a nutritionally-relevant health biomarker in honey bees (n = 9–12 colonies per diet treatment). **A** *vitellogenin* expression in colonies at each apiary site. **B** *Vitellogenin* expression in colonies fed the different diets. Error bars represent standard error (SE). Columns with different letters are significantly different at α = 0.05.

Honey bee colony nutrition is reliant on the production and conservation of Vitellogenin (Vg), an abdominal storage protein with important roles in brood rearing, stress responses, and overwintering [69, 70]. Colony *vg* mRNA expression was measured in pools of field-collected bees. Apiary site had a significant effect on *vg* expression (F _2, 81_ = 6.5, *P* = 0.0026) whereas feeding treatment did not (*P* > 0.05). Average colony *vg* expression at apiary site 1 was less than half of sites 2 and 3 (Fig. 9A). Pooled-bee *vg* expression has been shown to reflect colony performance and is positively correlated with brood levels, adult bee mass, and hive-stored pollen reserves [38–40]. We found that *vg* expression was primarily driven by apiary site, which agrees with similar analyses of field-collected bee samples [38–40]. The negative control and Ultra Bee diets produced the smallest colonies and trended towards the lowest *vg* levels (Fig. 9B). However, no significant relationship between *vg* levels and colony size was determined (*P* > 0.05).

### Gut microbiota abundance

Honey bees are populated by a simple and consistent microbiota with roles in nutrition and immune functions [71, 72]. The bee gut is dominated by gram-positive species, including two *Lactobacillus* taxa (Firm 5 and Firm 4) and *Bifidobacterium asteroides* [46, 73]. The gram negative species *Snodgrassella alvi* (Betaproteobacteria) and *Gilliamella apicola* (Gammaproteobacteria) are also considered “core” to the honey bee microbiota [74]. Nutrition modulates bee gut microbiota, which can have consequences on host immune function and pathogen susceptibility [44]. Microbiota of non-thriving colonies were previously shown to be depleted of health-promoting taxa such as *Lactobacillus* [75]. Here, we tested the effects of different artificial diets on honey bee gut microbiota abundance. Bacterial 16S rRNA abundances of prominent gut taxa were measured in a subset of colonies from each treatment group and at each apiary site (*n* = 9–12 colonies per diet treatment).

**Fig. 10:**
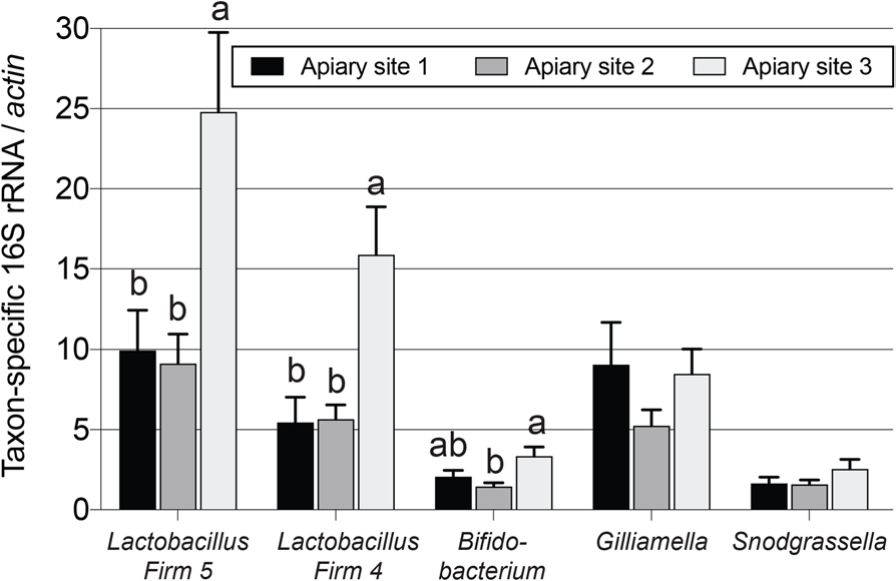
Relative gut microbiota bacterial 16S rRNA abundances in colonies at the different apiary sites (n = 29–30 colonies per apiary site). Error bars represent standard error (SE). For each taxon, columns with different letters are significantly different at α = 0.05.

**Fig. 11:**
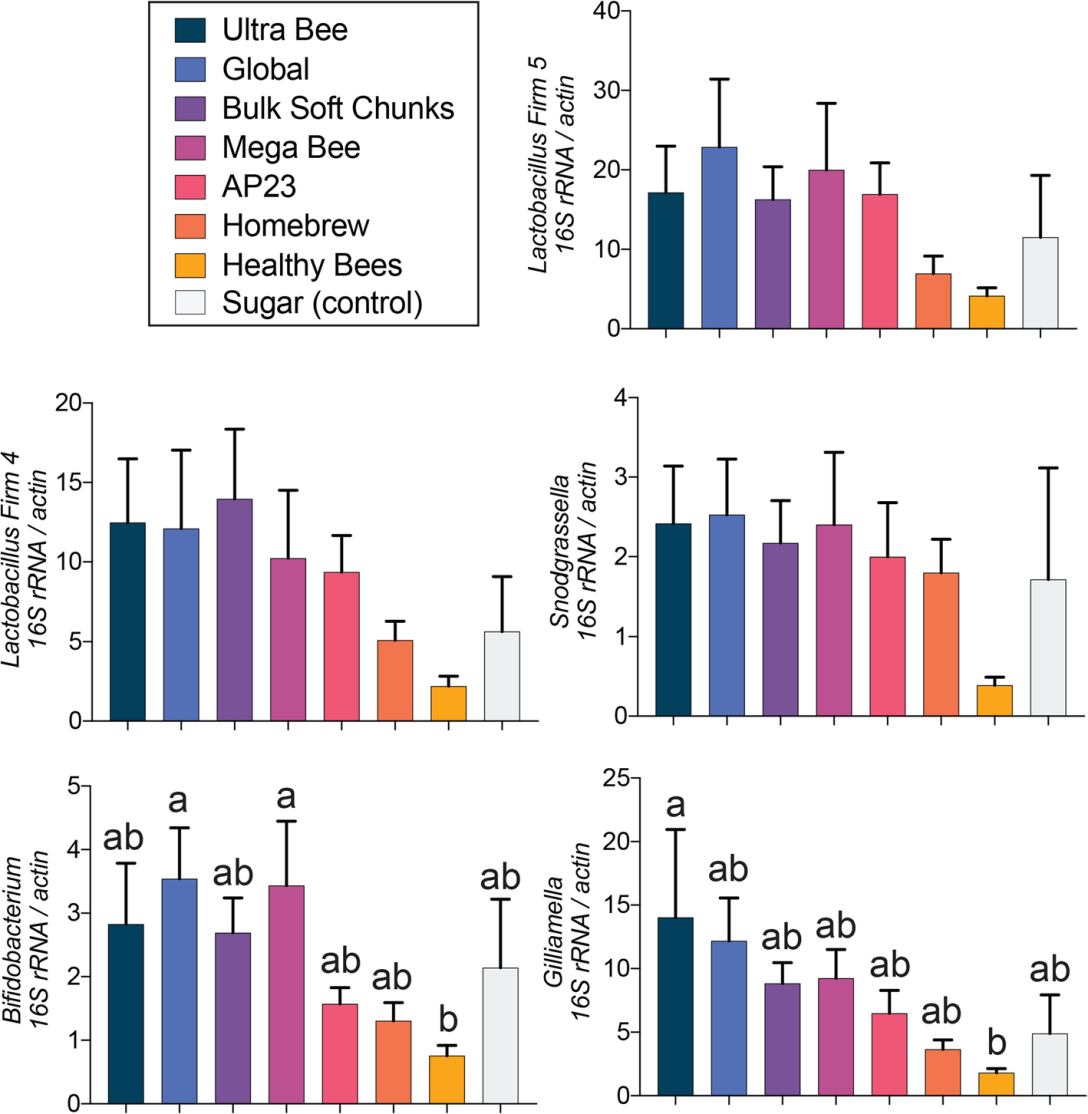
Relative gut microbiota bacterial 16S rRNA abundances in colonies fed the different diets (n = 9–12 colonies per diet treatment). Error bars represent standard error (SE). For each taxon, columns with different letters are significantly different at α = 0.05.

Apiary site had a significant impact on the abundance of *Lactobacillus* Firm 5 (F _2, 81_ = 6.5, *P* = 0.0028), *Lactobacillus* Firm 4 (F _2, 81_ = 8.9, *P* = 0.0004), and *Bifidobacterium* (F _2, 81_ = 6.0, *P* = 0.004). Colonies at apiary site 3 had the highest abundances of *Lactobacillus* Firm 5, *Lactobacillus* Firm 4, and *Bifidobacterium* (Fig. 10)*.* Even though the honey bee microbiota is highly consistent across populations, landscape exposure and environmental differences among sites play a role in the abundance of key taxa [76]. Diet had a significant impact on the abundance of *Bifidobacterium* (F _7, 81_ = 2.8, *P* = 0.0124) and *Gilliamella* (F _7, 81_ = 2.3, *P* = 0.026). Colonies fed the Healthy Bees diet had significantly lower *Bifidobacterium* and *Gilliamella* and trended towards the lowest abundances of the other taxa (Fig. 11).

Bacterial taxa were examined based on previously characterized nutrition and metabolic functions. *Lactobacillus* spp. and *Bifidobacterium* spp. are fermentative members of animal microbiota, including bees [77]. *Gilliamella apicola* has diverse carbohydrate utilization repertoires, including metabolism of toxic sugars present in pollen [78]. *Snodgrassella alvi* is non-fermentative but participates nutrient-sharing interactions with the fermentative bacterial community [79]. We hypothesized that variable diet compositions would differentially impact gut bacteria. However, apiary site overrode most diet effects and there was no clear relationship between microbiota abundance and colony size. Significantly reduced gut bacteria in colonies fed the Healthy Bees diet may represent a fitness disadvantage, which is consistent with reduced colony size and bee weights. A distinct feature of the Healthy Bee diet is the use of spirulina microalgae as a protein source (Table S2). Microalgae-based diets are generally effective pollen substitutes under laboratory conditions [43, 80] but more research is necessary to understand the effects of microalgae on bee colony health. Honey bee nutrition supplements commonly incorporate plant essential oils to improve attractiveness and spoilage characteristics (Table S2). The specific composition of essential oils in the Healthy Bees diet may have exerted an antimicrobial effect since essential oils are complex mixtures of compounds that can inhibit gut bacteria [82]. The prevalent use of essential oils in bee nutrition supplements warrants future investigation into their effects on gut microbiota.

### Pathogen levels

**Fig. 12:**
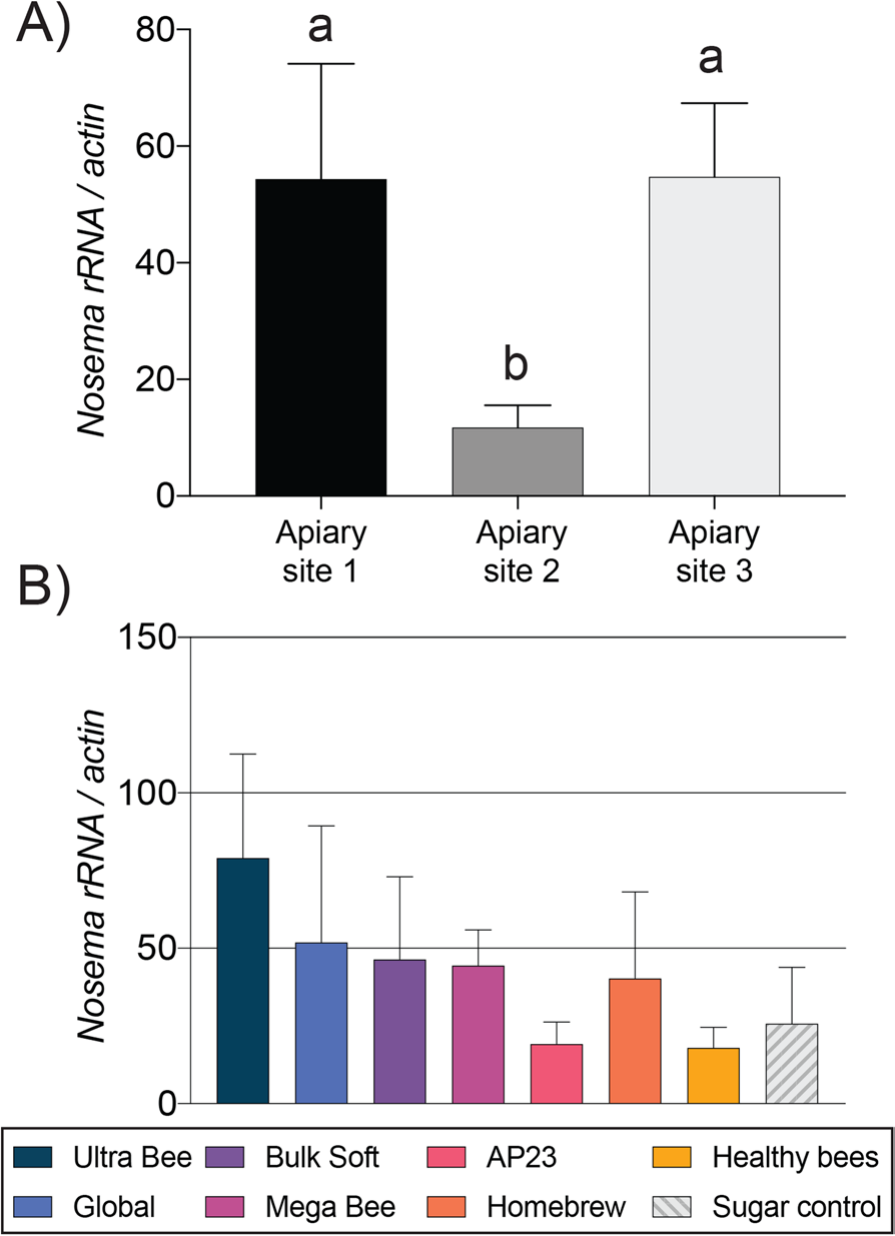
Relative rRNA abundance of the gut pathogen *Nosema* (n = 9–12 colonies per diet treatment). **A** *Nosema* abundance in honey bee colonies at each apiary site. **B** *Nosema* abundance in honey bee colonies fed the different diets. Error bars represent standard error (SE). Columns with different letters are significantly different at α = 0.05.

Numerous pathogens are linked to honey bee colony losses including the mite *Varroa destructor*, the microsporidia *Nosema ceranae*, and different RNA viruses. The *Varroa* mite is a vector of viral pathogens, in particular Deformed Wing Virus (DWV). In the absence of mites, DWV generally causes asymptomatic infection and is detectable in most colonies. *N. ceranae* is a microsporidian gut pathogen that also persists in most colonies at low levels of asymptomatic infection. Both DWV and *N. ceranae* have immunological and energetic costs that may interact with bee nutrition [7, 44, 83]. We monitored the abundances of these pathogens in subset of colonies sampled in November. Although previous studies have shown that pollen-rich diets stimulate *N. ceranae* abundance [84], we found that *N. ceranae* levels were significantly influenced by apiary site but not by diet treatment (F _2, 81_ = 3.4, *P* = 0.0371; Fig. 12). Increased *N. ceranae* levels may be associated with a reduction of core gut microbiota [44]. However, there was not a significant relationship between *N. ceranae* levels and gut microbiota abundance (*P* > 0.05).

Deformed Wing Virus levels were not significantly impacted by apiary site or diet treatment (*P* > 0.05; Fig. S4).

## Conclusions

Despite the prevalent use of honey bee nutrition supplements, there is limited and sometimes conflicting information regarding their benefits in large-scale field applications. This is likely due to variations in local apiary site environments, including the amount and type of natural pollen forage available at the time of testing. In general, protein-containing diets led to larger colonies than the negative control diet. In the context of this study, macronutrient content alone could not be attributed to diet effects. However, dietary essential amino acid deficiencies relative to leucine were strongly correlated with colony size and average bee weight. This suggests that optimization of EAA balance could improve protein synthesis by maximizing leucine utilization. Future experiments will aim to test this hypothesis in laboratory and field settings using controlled diet formulations.

Diet effectiveness is generally attributed to the nutrient levels present rather than their origin. Since this study mainly focused on commercial diet formulas, it is possible that some diets could have provided an overlooked nutritional factor not present in the other diets such as essential micronutrients. Notably, the Global and Homebrew diets contained pollen, the bee’s natural food source. Nevertheless, two of the five artificial diets produced significantly larger colonies than the negative control. Further development is necessary in order to reproduce the effects of pollen nutrition, although some completely artificial diets were effective in the context of our study.

In conclusion, the present study showed that artificial feed can improve colony performance and health of commercially managed honey bees. Our results support the notion that longitudinal studies are necessary to understand honey bee colony nutritional requirements and the effects of supplemental feeding. For many regions in the US, the fall and winter months leading up to pollination of spring crops are marked by a reduction in floral resources available to bees. Feeding regimens tailored to specific beekeeping operations and management goals are likely to provide the most benefits. Continued improvement of bee diets has the potential to improve feed costs and increase pollination efficiency by supporting larger, healthier colonies.

## Supplementary Information

**Additional file 1: Table S1.** Quantitative PCR primers used in this study. **Table S2.** List of diet ingredients provided by the manufacturers. **Figure S1**. **A)** Suggested proportions of essential amino acids according to honey bee growth requirements established by de Groot 1953. **B)** Leucine percentage of total EAAs in the tested diets. **Figure S2.** Protein to lipid ratios of the test diets. **Figure S3.** Normalization of dietary EAA content to leucine. **Figure S4.** Relative Deformed Wing Virus A (DWVA) abundance in colonies fed the different diets.

## Data Availability

The datasets used and/or analyzed in this study are available from the corresponding author on reasonable request.

## References

[1] Kulhanek K, Steinhauer N, Rennich K, Caron DM, Sagili RR, Pettis JS (2017). A national survey of managed honey bee 2015–2016 annual colony losses in the USA. J Apic Res.

[2] Traynor KS, Rennich K, Forsgren E, Rose R, Pettis J, Kunkel G (2016). Multiyear survey targeting disease incidence in US honey bees. Apidologie..

[3] Johnson RM, Ellis MD, Mullin CA, Frazier M (2010). Pesticides and honey bee toxicity – USA. Apidologie..

[4] López-Uribe MM, Ricigliano VA, Simone-Finstrom M (2020). Defining pollinator health: a holistic approach based on ecological, genetic, and physiological factors. Annu Rev Anim Biosci.

[5] Dolezal AG, Toth AL (2018). Feedbacks between nutrition and disease in honey bee health. Curr Opin Insect Sci.

[6] Vodovnik C, Borshagovski AM, Hakala SM, Leponiemi M, Freitak D (2021). Coeffects of diet and neonicotinoid exposure on honeybee mobility and food choice. Apidologie..

[7] DeGrandi-Hoffman G, Chen Y (2015). Nutrition, immunity, and viral infections in honey bees. Curr Opin Insect Sci.

[8] Tritschler M, Vollmann JJ, Yanez O, Chejanovsky N, Crailsheim K, Neumann P. Protein nutrition governs within-host race of honey bee pathogens. Sci Rep. 2017;7:1–11. 10.1038/s41598-017-15358-w PMC5678143 29118416

[9] Koch H, Brown MJ, Stevenson PC (2017). The role of disease in bee foraging ecology. Curr Opin Insect Sci.

[10] Gong Y, Diao Q (2017). Current knowledge of detoxification mechanisms of xenobiotic in honey bees. Ecotoxicology..

[11] Di Pasquale G, Salignon M, Le Conte Y, Belzunces LP, Decourtye A, Kretzschmar A (2013). Influence of pollen nutrition on honey bee health: do pollen quality and diversity matter?. PLoS One.

[12] Di Pasquale G, Alaux C, Le Conte Y, Odoux JF, Pioz M, Vaissière BE (2016). Variations in the availability of pollen resources affect honey bee health. PLoS One.

[13] Brodschneider R, Crailsheim K (2010). Nutrition and health in honey bees. Apidologie..

[14] Crailsheim K (1990). The protein balance of the honey bee worker. Apidologie..

[15] Huang Z (2012). Pollen nutrition affects honey bee stress resistance. Terr Arthropod Rev.

[16] Alaux C, Dantec C, Parrinello H, Le Conte Y (2011). Nutrigenomics in honey bees: digital gene expression analysis of pollen's nutritive effects on healthy and varroa-parasitized bees. BMC Genomics.

[17] Meikle WG, Weiss M, Maes PW, Fitz W, Snyder LA, Sheehan T (2017). Internal hive temperature as a means of monitoring honey bee colony health in a migratory beekeeping operation before and during winter. Apidologie..

[18] Roulston TH, Cane J, Buchmann S (2000). What governs protein content of pollen: pollinator preferences, pollen–pistil interactions, or phylogeny?. Ecol Monogr.

[19] Roulston TH, Cane JH. Pollen nutritional content and digestibility for animals. Plant Syst Evol. 2000;222:187–209.

[20] Donkersley P, Rhodes G, Pickup RW, Jones KC, Wilson K (2014). Honeybee nutrition is linked to landscape composition. Ecol Evol.

[21] Sponsler DB, Johnson RM (2015). Honey bee success predicted by landscape composition in Ohio, USA. Peer J.

[22] Dolezal AG, Carrillo-Tripp J, Miller WA, Bonning BC, Toth AL (2016). Intensively cultivated landscape and varroa mite infestation are associated with reduced honey bee nutritional state. PLoS One.

[23] Taylor B. Threats to an ecosystem service: pressures on pollinators. Front Ecol Environ. 2013;11:1–9.

[24] Bartomeus I, Ascher J, Wagner D, Danforth B, Colla S, Kornbluth S (2011). Climate-associated phenological advances in bee pollinators and bee-pollinated plants. PNAS..

[25] Settele J, Bishop J, Potts S (2016). Climate change impacts on pollination. Nat Plants.

[26] Mortensen AN, Jack CJ, Bustamante TA, Schmehl DR, Ellis JD (2019). Effects of supplemental pollen feeding on honey bee (hymenoptera: Apidae) colony strength and Nosema spp. infection. J Econ Entomol.

[27] Lamontagne-Drolet M, Samson-Robert O, Giovenazzo P, Fournier V (2019). The impacts of two protein supplements on commercial honey bee (*Apis mellifera L.*) colonies. J Apic Res.

[28] Mattila HR, Otis GW (2006). Influence of pollen diet in spring on development of honey bee (hymenoptera: Apidae) colonies. J Econ Entomol.

[29] Noordyke ER, Ellis JD (2021). Reviewing the efficacy of pollen substitutes as a management tool for improving the health and productivity of western honey bee (*Apis mellifera*) colonies. Front Sustain Food Syst.

[30] De Groot AP (1953). Protein and amino acid requirements of the honeybee (*Apis mellifica L*.). Physiol comp. Oecol..

[31] Avni D, Hendriksma HP, Dag A, Uni Z, Shafir S (2014). Nutritional aspects of honey bee-collected pollen and constraints on colony development in the eastern Mediterranean. J Insect Physiol.

[32] Arien Y, Dag A, Zarchin S, Masci T, Shafir S (2015). Omega-3 deficiency impairs honey bee learning. PNAS..

[33] Wegener J, Jakop U, Schiller J, Müller K (2018). The membrane phospholipid composition of honeybee (*Apis mellifera*) workers reflects their nutrition, fertility, and vitellogenin stores. Insect Soc.

[34] Standifer LN, Haydak MH, Mills JP, Levin MD. Influence of pollen in artificial diets on food consumption and brood production in honey bee colonies. Am Bee J. 1973;113:94–5.

[35] Alqarni AS (2006). Influence of some protein diets on the longevity and some physiological conditions of honeybee *Apis mellifera L*. workers. Res. J Biol Sci.

[36] Sheesley B, Poduska B (1970). Strong honeybee colonies prove value in almond pollination. Calif Agric.

[37] Goodrich BK (2019). Do more bees imply higher fees? Honey bee colony strength as a determinant of almond pollination fees. Food Policy.

[38] Ricigliano VA, Mott BM, Floyd AS, Copeland DC, Carroll MJ, Anderson KE (2018). Honey bees overwintering in a southern climate: longitudinal effects of nutrition and queen age on colony-level molecular physiology and performance. Sci Rep.

[39] Alaux C, Allier F, Decourtye A, Odoux JF, Tamic T, Chabirand M, et al. “Landscape physiology” approach for assessing bee health highlights the benefits of floral landscape enrichment and semi-natural habitats. Sci Rep. 2017;7:1–10. 10.1038/srep40568 PMC5234012 28084452

[40] Ricigliano VA, Mott BM, Maes PW, Floyd AS, Fitz W, Copeland DC (2019). Honey bee colony performance and health are enhanced by apiary proximity to US conservation reserve program (CRP) lands. Sci Rep.

[41] Azzouz-Olden F, Hunt A, DeGrandi-Hoffman G (2018). Transcriptional response of honey bee (*Apis mellifera*) to differential nutritional status and *Nosema* infection. BMC Genomics.

[42] Corby-Harris V, Jones BM, Walton A, Schwan MR, Anderson KE (2014). Transcriptional markers of sub-optimal nutrition in developing *Apis mellifera* nurse workers. BMC Genomics.

[43] Ricigliano VA, Simone-Finstrom M (2020). Nutritional and prebiotic efficacy of the microalga *Arthrospira platensis* (spirulina) in honey bees. Apidologie..

[44] Castelli L, Branchiccela B, Garrido M, Invernizzi C, Porrini M, Romero H (2020). Impact of nutritional stress on honeybee gut microbiota, immunity, and *Nosema ceranae* infection. Microb Ecol.

[45] Ricigliano VA, Anderson KE (2020). Probing the honey bee diet-microbiota-host axis using pollen restriction and organic acid feeding. Insects..

[46] Anderson KE, Ricigliano VA (2017). Honey bee gut dysbiosis: a novel context of disease ecology. Curr Opin Insect Sci.

[47] Kwong WK, Mancenido AL, Moran NA (2017). Immune system stimulation by the native gut microbiota of honey bees. R Soc Open Sci.

[48] Kenneth H. Official methods of analysis of the Association of Official Analytical Chemists. 15th ed: The Association; 1990.

[49] American Association of Cereal Chemists, Approved Methods Committee. Approved methods of the American Association of Cereal Chemists: AACC; 2000.

[50] Livak KJ, Schmittgen TD. Analysis of relative gene expression data using real-time quantitative PCR and the 2(−Delta Delta C(T)) method. Methods. 2001;25:402–8. 10.1006/meth.2001.1262 11846609

[51] DeGrandi-Hoffman G, Chen Y, Huang E, Huang MH (2010). The effect of diet on protein concentration, hypopharyngeal gland development and virus load in worker honey bees (*Apis mellifera L*.). J Insect Physiol.

[52] De Jong D, da Silva EJ, Kevan PG, Atkinson JL (2015). Pollen substitutes increase honey bee haemolymph protein levels as much as or more than does pollen. J Apic Res.

[53] Li C, Xu B, Wang Y, Feng Q, Yang W (2012). Effects of dietary crude protein levels on development, antioxidant status, and total midgut protease activity of honey bee (*Apis mellifera ligustica*). Apidologie..

[54] Crone MK, Grozinger CM. Pollen protein and lipid content influence resilience to insecticides in honey bees (*Apis mellifera*). J Exp Biol. 2021;224:jeb.242040. 10.1242/jeb.242040 33758024

[55] Arien Y, Dag A, Yona S, Tietel Z, Cohen TL, Shafir S (2020). Effect of diet lipids and omega-6 3 ratio on honey bee brood development, adult survival and body composition. J Insect Physiol.

[56] Arrese EL, Soulages JL (2010). Insect fat body: energy, metabolism, and regulation. Annu Rev Entomol.

[57] Ischebeck T (2016). Lipids in pollen - they are different. BBA..

[58] Chakrabarti P, Morré JT, Lucas HM, Maier CS, Sagili RR (2019). The omics approach to bee nutritional landscape. Metabolomics.

[59] Hulbert AJ, Turner N, Storlien LH, Else PL (1999). Dietary fats and membrane function: implications for metabolism and disease. Biol Rev Camb Philos Soc.

[60] Morrison KE, Jašarević E, Howard CD, Bale TL (2020). It's the fiber, not the fat: significant effects of dietary challenge on the gut microbiome. Microbiome..

[61] Bonvehi SJ, Jorda RE (1997). Nutrient composition and microbiological quality of honeybee-collected pollen in Spain. J Agric Food Chem.

[62] Doull K. Effects of attractants and phagostimulants in pollen and pollen supplement on the feeding behaviour of honeybees in the hive. J Apic Res. 1974;13:47–54.

[63] Burroughs EW, Burroughs H, Mitchell H (1940). The interdependence among amino acids in their utilization in the endogenous metabolism. J Nutr.

[64] Robinson FA, Nation J (1966). Artificial diets for honey bees, *Apis mellifera*. Fla Entomol.

[65] Smart M, Pettis J, Rice N, Browning Z, Spivak M (2016). Linking measures of colony and individual honey bee health to survival among apiaries exposed to varying agricultural land use. PLoS One.

[66] Crailsheim K, Schneider L, Hrassnigg N (1992). Pollen consumption and utilization in worker honeybees (*Apis mellifera carnica*): dependence on individual age and function. J Insect Physiol.

[67] Noordyke ER, van Santen E, Ellis JD (2021). Tracing the fate of pollen substitute patties in western honey bee (hymenoptera: Apidae) colonies. J Econ Entomol.

[68] Hendriksma HP, Pachow CD, Nieh JC (2019). Effects of essential amino acid supplementation to promote honey bee gland and muscle development in cages and colonies. J Insect Physiol.

[69] Amdam GV, Norberg K, Hagen A, Omholt SW (2003). Social exploitation of vitellogenin. Proc Natl Acad Sci.

[70] Amdam GV, Norberg K, Omholt SW, Kryger P, Lourenço AP, Bitondi MMG (2005). Higher vitellogenin concentrations in honey bee workers may be an adaptation to life in temperate climates. Insect Soc.

[71] Raymann K, Moran NA (2018). The role of the gut microbiome in health and disease of adult honey bee workers. Curr Opin Insect Sci.

[72] Kwong WK, Moran NA (2016). Gut microbial communities of social bees. Nature.

[73] Kwong WK, Engel P, Koch H, Moran NA (2014). Genomics and host specialization of honey bee and bumble bee gut symbionts. PNAS..

[74] Kwong WK, Moran NA (2013). Cultivation and characterization of the gut symbionts of honey bees and bumble bees: description of *Snodgrassella alvi* gen. Nov., sp. nov., a member of the family *Neisseriaceae* of the *Betaproteobacteria*, and *Gilliamella apicola* gen. Nov., sp. nov., a member of *Orbaceae* fam. Nov., Orbales Ord. Nov., a sister taxon to the order “Enterobacteriales” of the Gammaproteobacteria. Int J Syst Evol Microbiol.

[75] Ribière C, Hegarty C, Stephenson H, Whelan P, O'Toole PW (2019). Gut and whole-body microbiota of the honey bee separate thriving and non-thriving hives. Microb Ecol.

[76] Jones JC, Fruciano C, Hildebrand F, Toufalilia A, Balfour NJ, Bork P (2018). Gut microbiota composition is associated with environmental landscape in honey bees. Ecol Evol.

[77] Bottacini F, Milani C, Turroni F, Sanchez B, Foroni E, Duranti S (2012). *Bifidobacterium asteroides* PRL2011 genome analysis reveals clues for colonization of the insect gut. PLoS One.

[78] Zheng H, Nishida A, Kwong WK, Koch H, Engel P, Steele MI (2016). Metabolism of toxic sugars by strains of the bee gut symbiont *Gilliamella apicola*. mBio..

[79] Kešnerová L, Mars R, Ellegaard KM, Troilo M, Sauer U, Engel P (2017). Disentangling metabolic functions of bacteria in the honey bee gut. PLoS Biol.

[80] Ricigliano VA, Ihle KE, Williams ST (2021). Nutrigenetic comparison of two Varroa-resistant honey bee stocks fed pollen and spirulina microalgae. Apidologie..

[81] Ricigliano VA (2020). Microalgae as a promising and sustainable nutrition source for managed honey bees. Arch Insect Biochem Physiol.

[82] Cao Y, Liu H, Qin N, Ren X, Zhu B, Xia X (2020). Impact of food additives on the composition and function of gut microbiota: a review. Trends Food Sci Technol.

[83] Mayack C, Naug D (2009). Energetic stress in the honeybee *Apis mellifera* from *Nosema ceranae* infection. J Invertebr Pathol.

[84] Basualdo M, Barragán S, Antúnez K (2014). Bee bread increases honeybee haemolymph protein and promote better survival despite of causing higher *Nosema ceranae* abundance in honeybees. Environ Microbiol Rep.

