# Supplementary material for: Effects of different artificial diets on commercial honey bee colony performance, health biomarkers, and gut microbiota
Source: BMC Vet Res. 2022 Jan 21;18:52. doi: 10.1186/s12917-022-03151-5 (PMC8780706; doi:10.1186/s12917-022-03151-5)
Supplement: Supplementary file 1 — Additional file 1: Table S1. Quantitative PCR primers used in this study. Table S2. List of diet ingredients provided by the manufacturers. Figure S1. A) Suggested proportions of essential amino acids according to honey bee growth requirements established by de Groot 1953. B) Leucine percentage of total EAAs in the tested diets. Figure S2. Protein to lipid ratios of the test diets. Figure S3. Normalization of dietary EAA content to leucine. Figure S4. Relative Deformed Wing Virus A (DWVA) abundance in colonies fed the different diets. [file 12917_2022_3151_MOESM1_ESM.docx]

| **Gene**  **(accession number)** | **Forward 5’-3’** | **Reverse 5’-3’** | **Annealing temperature (°C)** | **Study** |
| --- | --- | --- | --- | --- |
| *actin*  (XM_623378) | TGCCAACACTGTCCTTTCTG | AGAATTGACCCACCAATCCA | 55.0 | (Alaux et al., 2011) |
| *vitellogenin (vg)*  (AJ517411) | GTTGGAGAGCAACATGCAGA | TCGATCCATTCCTTGATGGT | 57.5 | (Salmela et al., 2016) |
| *Lactobacillus*  *Firm 5 16S rRNA*  (JX099547) | GCAACCTGCCCTWTAGCTTG | GCCCATCCTKTAGTGACAGC | 60.0 | (Kešnerová et al., 2017) |
| *Lactobacillus*  *Firm 4 16S rRNA*  (DQ837632) | AGTCGAGCGCGGGAAGTCA | AGCCGTCTTTCAACCAGCACT | 60.0 | (Kešnerová et al., 2017) |
| *Bifidobacterium 16S rRNA*  (AB437355) | ATGCAAGTCGAACGGGATCC | CATCCCATRCCGGTAAACCC | 60.0 | (Kešnerová et al., 2017) |
| *Gilliamella 16S rRNA*  (JQ936674) | CTTTGTTGCCATCGGTTAGGCC | CCGCTTGCTCTCGCGAGG | 60.0 | (Kešnerová et al., 2017) |
| *Nosema ceranae* rRNA  (DQ486027) | AAGAGTGAGACCTATCAGCTAGTTG | CCGTCTCTCAGGCTCCTTCTC | 58.0 | (Bourgeois et al., 2010) |
| Deformed wing virus (DWVA)  (AY292384.1) | GAGATTGAA GCGCATGAACA | TGAATTCAG TGTCGCCCA TA | 60.0 | (Boncristiani et al., 2012) |

**Table S1.** Quantitative PCR primers used in this study

| **Diet** | **Manufacturer** | **Ingredients** |
| --- | --- | --- |
| Global | Global Patties | sugar, soy, yeast, lipid mix, vitamins, minerals, and sterilized wildflower pollen (15% by dry weight) |
| Ultra Bee | Mann Lake Ltd. | plant protein, high fructose corn syrup, sugar, canola oil, soybean oil, palm oil, lemongrass oil, spearmint extract, probiotics, wheat flour, vitamins, and minerals |
| Bulk Soft | Mann Lake Ltd. | plant protein products, fructose, processed grain by-products, dextrose, sugar, water, canola oil, vegetable shortening, lemongrass oil, spearmint oil, lecithin, thymol, sodium chloride, vitamin A, vitamin D3, vitamin E, ascorbic acid, zinc, menadione, riboflavin, pyridoxine, folic acid, and biotin |
| Mega Bee | Castle Dome Solutions | plant protein products, sugar, processed grain by-products, citric acid, potassium sorbate, sodium propionate |
| AP23 | Dadant | plant protein products, sugar, grain products, dried egg product, lecithin, dl-methionine, potassium chloride, choline chloride, natural flavoring, mixed tocopherols, d-alpha tocopheryl acetate, ascorbic acid |
| Homebrew | Custom formulation (this study) | Soy isolate, soy flour, yeast, sugar, canola oil, lysine, zinc, essential oils, and pollen (20% by dry weight) |
| Healthy Bees | Healthy Bees LLC | spirulina, corn gluten, brewer's yeast, dextrose, invert sucrose syrup, coconut oil, canola oil, essential oils, natural plant extracts |

**Table S2.** List of diet ingredients provided by the manufacturers

**Figure S1**. **A)** Suggested proportions of essential amino acids according to honey bee growth requirements established by de Groot 1953. **B)** Leucine percentage of total EAAs in the tested diets.

**Figure S2.** Protein to lipid ratios of the test diets.

**Figure S3.** Normalization of dietary EAA content to leucine.

**Figure S4.** Relative Deformed Wing Virus A (DWVA) abundance in colonies fed the different diets.
